# Immune‐related adverse events in the treatment of Hodgkin lymphoma with immune checkpoint inhibitors

**DOI:** 10.1111/bjh.70402

**Published:** 2026-02-26

**Authors:** Lisa Argnani, Vittorio Stefoni, Beatrice Casadei, Carla Pelusi, Valentina Lo Preiato, Uberto Pagotto, Anna Buganè, Cinzia Pellegrini, Alessandro Broccoli, Pier Luigi Zinzani

**Affiliations:** ^1^ Dipartimento di Scienze Mediche e Chirurgiche (DIMEC) Alma Mater Studiorum Università of Bologna Bologna Italy; ^2^ IRCCS Azienda Ospedaliero‐Universitaria di Bologna Istituto di Ematologia “Seràgnoli” Bologna Italy; ^3^ Division of Endocrinology and Diabetes Prevention and Care IRCCS Azienda Ospedaliero‐Universitaria di Bologna Bologna Italy

**Keywords:** Hodgkin lymphoma, immune checkpoint inhibitors, immune‐related adverse events

## Abstract

Immune checkpoint inhibitors (ICIs) have shown to be effective in the treatment of Hodgkin lymphoma (HL). ICI treatment is associated with the occurrence of a series of immune‐related adverse events (irAEs). We conducted an ambispective observational study on 96 patients with relapsed/refractory HL treated with ICIs to determine the incidence of irAEs, their type, severity and their correlation with treatment outcomes. Fifty patients were treated with pembrolizumab (52.1%) and 46 patients were treated with nivolumab (47.9%). A total of 22 patients (22.9%) developed at least one irAE, resulting in a total of 27 irAEs. The results of our study did not show statistically significant differences between the two cohorts of patients, suggesting that the presence of these peculiar toxicities does not significantly impact outcomes and survivals in patients with HL treated with ICIs. However, the complexity of irAEs, their classification as such and the variability in response to immunotherapy require further studies.

## INTRODUCTION

In 2020, a total of 83 087 new cases of Hodgkin lymphoma were reported. The global age‐standardized incidence rate was 0.98 per 100 000 people and is defined by its distinct pathological features consistent with Reed Sternberg cells in a background of inflammatory cells, which enable the evasion of immune‐mediated anti‐tumour mechanisms.^1^, ^2^, ^3^ Although most patients with classical HL (cHL) can be cured with standard chemo(radio)therapy, patients who are unresponsive to initial treatment or who are not candidates for curative regimens have poor outcomes.^4^, ^5^, ^6^ Approximately 10%–25% of patients with advanced or early‐stage unfavourable risk disease have disease relapse.^6^ The standard of care with salvage chemotherapy followed by autologous stem cell transplantation (ASCT) cures only about 50% of patients.^7^, ^8^, ^9^ Older patients and those with medical comorbidities remain at a particularly high risk for relapse and inferior survival.^10^ With modern front‐line treatment regimens, patients over the age of 60 years had about a 15% worse 3‐year progression‐free survival (PFS) compared with younger patients.^10^

Immune checkpoint inhibitors (ICIs, namely, pembolizumab and nivolumab) enable the reconstitution of the immune response to target malignant cells, offering an alternative mechanism of action from traditional cytotoxic chemotherapy and have thus transformed the treatment landscape for relapsed/refractory (R/R) cHL.^11^

ICIs can reactivate the immune response, making it more effective and enhancing the immune system's ability to recognize and destroy neoplastic cells.^12^ In patients with R/R HL, the clinical results obtained with ICI treatment have been encouraging, with significant outcomes even in cases of disease refractory to conventional chemotherapy. In several clinical studies, high overall response rate (ORR) and duration of response (DoR) have been observed, with a positive impact on overall survival (OS) and PFS, allowing for tangible improvements in clinical outcomes.^13^, ^14^, ^15^, ^16^, ^17^, ^18^, ^19^

However, the use of ICIs is not without complications. The so‐called immune‐related AEs (irAEs) are peculiar class of AEs, resulting from excessive and uncontrolled activation of the immune system, which represents a significant issue in treatment with these drugs.^20^, ^21^, ^22^ In fact, irAEs present with greater heterogeneity than AEs of the other conventional drugs, making their diagnosis more complex.^20^, ^23^ Indeed, irAEs can involve a wide range of organs and tissues, with manifestations ranging from mild and transient forms to serious complications, sometimes fatal.^20^, ^23^ Some research has suggested a potential correlation between the onset of irAEs and an enhanced anti‐tumour response, with most of the data coming from Oncology.^20^, ^23^ After the first part of our research on the relationship between ICIs, irAEs and non‐Hodgkin lymphoma, we explored our experience in R/R cHL to further address these issues, poorly documented in haematological literature.^24^, ^25^, ^26^, ^27^

## MATERIALS AND METHODS

An observational ambispective study was conducted on patients with R/R cHL treated with ICIs at our Institution in 2014–2022. The study was conducted in a real‐life context; thus, the two drugs were administered as per official approval indications, that is, pembrolizumab from third line and beyond and nivolumab from fourth line and beyond. The study was approved by our local Ethical Committee (Comitato Etico Area Vasta Emilia Centrale di Bologna, IRCCS Azienda Ospedaliero‐Universitaria di Bologna, approval id code 730/2019/Oss/AOUBo) and registered in the Italian Registry of Observational Studies. Patients provided signed informed consent, as applicable, in accordance with the Declaration of Helsinki.

Primary objective of the study was to determine the incidence of irAEs in patients affected by R/R HL undergoing ICIs treatment, also assessing the type, severity and timing of onset, management, outcome and relationship with study drugs of these events. Patients remained in follow‐up till the resolution of irAEs. A minimum of 12 months of follow‐up was required for the analyses to evaluate late irAEs, when applicable.

Secondary objectives were activity and disease control of ICIs along with their relationship with irAEs onset. End‐points were ORR (sum of complete response [CR] and partial response [PR] at the end of treatment and before any type of consolidation), best response rate reached at any time, OS, PFS, disease‐free survival (DFS), DoR and safety.^28^ Response was assessed using the International Working Group revised response criteria for malignant lymphoma.^28^, ^29^ Safety and tolerability were evaluated by recording incidence, severity and type of any AE according to ASCO recommendations.^20^, ^23^

Demographics and patients' characteristics as well as AEs were summarized by descriptive statistics. Continuous variables were reported as median (range) for non‐normally distributed data and compared using the Student's *t*‐test or Mann–Whitney *U*‐test. Categorical variables were reported as absolute and relative frequencies and compared using Fisher's exact test or chi‐squared test, as applicable. Correlations were tested among irAEs occurrence, effectiveness of ICIs and patients' survivals. Survival functions were estimated by using the Kaplan–Meier method with their relative 95% confidence interval (CI), the comparison of survival curves by using the log‐rank test (Mantel–Cox) and the Gehan–Breslow–Wilcoxon test, as applicable.

Statistical analyses were performed with Stata 17 (StataCorp LP, TX) and *p* values were set at 0.05.

## RESULTS

### Study population

The study included a total of 96 patients with relapsed/refractory HL, diagnosed between 1999 and 2021, who underwent treatment with ICIs. The characteristics of the enrolled patients are summarized in Table 1.

**TABLE 1:** Patients’ characteristics.

Characteristics, *n* (%)	Total (*N* = 96)	No irAE (*N* = 55)	irAE (*N* = 41)	*p*
Sex				0.534
M	55 (57.3)	33 (60.0)	22 (53.7)	
F	41 (42.7)	22 (40.0)	19 (46.3)	
Age, median years	38.2	36.8	38.4	0.080
Stage				0.332
I	0 (0)	0 (0)	0 (0)	
II	40 (41.7)	22 (40.0)	18 (43.9)	
III	17 (17.7)	8 (14.5)	9 (22.0)	
IV	35 (36.4)	21 (38.2)	14 (34.1)	
NA	4 (4.2)	4 (7.3)	0 (0)	
Extranodal site				0.768
Yes	40 (41.7)	24 (43.6)	16 (39.1)	
No	56 (58.3)	31 (56.4)	25 (60.9)	
B symptoms				0.491
No	53 (34.4)	19 (34.6)	14 (34.1)	
Yes	43 (44.8)	36 (65.4)	27 (65.9)	
Bulky				0.147
Yes	28 (29.2)	19 (34.6)	9 (22.0)	
No	68 (70.8)	36 (65.4)	32 (78.0)	
Final response after first line				0.064
CR	26 (27.1)	16 (29.1)	10 (24.4)	
SD	6 (6.2)	1 (1.8)	5 (12.2)	
PD	55 (57.3)	35 (63.6)	20 (48.8)	
PR	9 (9.4)	3 (5.5)	6 (14.6)	
Status pre‐ICI				0.686
Refractory	74 (77.1)	42 (76.4)	32 (78.0)	
Relapsed	21 (21.9)	12 (21.8)	9 (22.0)	
Consolidation	1 (1.0)	1 (1.8)	0 (0)	
Therapies pre‐ICI				0.133
2	17 (17.7)	7 (12.7)	10 (24.4)	
3	41 (42.7)	29 (52.7)	12 (29.3)	
4	17 (17.7)	9 (16.4)	8 (19.5)	
5	11 (11.5)	6 (10.9)	5 (12.3)	
>5	10 (10.4)	4 (7.3)	6 (14.6)	
Prior brentuximab vedotin	62 (64.6)	46	16	0.002
Prior radiation	25 (26.0)	21	4	0.003
Prior ASCT	43 (44.8)	31	12	0.092
Response to the last therapy prior to ICI				0.395
SD	7 (7.3)	4 (7.3)	3 (7.3)	
PD	64 (66.7)	37 (67.3)	27 (65.9)	
CR	16 (16.6)	11 (20.0)	5 (12.2)	
PR	9 (9.4)	3 (5.4)	6 (14.6)	
Stage pre‐ICI				0.142
I	0 (0)	0 (0)	0 (0)	
II	22 (22.9)	15 (27.3)	7 (17.1)	
III	23 (24.0)	10 (18.2)	13 (31.7)	
IV	32 (33.3)	16 (29.1)	16 (39.0)	
NA	19 (19.8)	14 (25.4)	5 (12.2)	
B Symptoms pre‐ICI				0.469
Yes	9 (9.4)	5 (9.1)	4 (9.7)	
No	87 (90.6)	50 (90.9)	37 (90.3)	
ECOG PS score				0.635
0	86 (89.6)	51 (92.7)	35 (85.4)	
1	9 (9.4)	4 (7.3)	5 (12.2)	
2	1 (1.0)	0 (0)	1 (2.4)	
ICI				0.366
Pembrolizumab	50 (52.1)	27 (49.1)	23 (56.1)	
Nivolumab	45 (47.9)	28 (50.9)	17 (43.9)	

Abbreviations: ASCT, autologous stem cell transplantation; CR, complete response; ECOG PS, Eastern Cooperative Oncology Group performance status; ICI, immune checkpoint inhibitors; irAE, immune‐related adverse event; NA, not available; PR, partial response; SD, stable disease; PD, progression of disease.

The distribution by gender showed a male prevalence, with 55 male patients (57.3%) and 41 female patients (42.7%). The median age at diagnosis was found to be 30 years (range 19–61). Regarding the stage of disease at diagnosis, the data indicate some variability among the analysed subjects, with a predominance of patients in advanced stages. The presence of extranodal sites was documented in 41.7% of cases. In this study, patients underwent various therapeutic regimens before the administration of ICIs therapy. The most commonly used initial therapeutic regimen was the Adriamycin, Bleomycin, Vinblastine, and Dacarbazine protocol, employed in 85.4% of patients. The median number of therapeutic lines received before the introduction of ICIs was 3 (range 2–16). Among the ICIs used, pembrolizumab was administered to 50 patients (52.1%) and nivolumab to 46 patients (47.9%). We followed indications coming from formal approval in Italy for HL, that is, pembrolizumab from third line and nivolumab from fourth line.

No differences were detected between the two patients' cohorts, in both medical history and at baseline, except for the previous use of brentuximab vedotin and radiotherapy, differences due to the wide time range of the sample considered.

Patients received an average of five cycles of treatment with ICIs (range 1–52), without significant dose reductions. Seventy‐six patients discontinued treatment early: 38 due to progression of disease (PD), 9 for bridge to ASCT, 10 for bridge to allogeneic transplantation, 9 for CR, 2 for stable disease, 6 due to AEs (LDH increase, grade 4 hepatic toxicity, persistent eosinophilia, acute interstitial nephritis, renal toxicity, pancreatitis), 1 at patient's request to stop treatment against the doctor's advice and 1 due to the patient's death. Only one irAE, that is, acute interstitial nephritis, caused treatment discontinuation. All treatment interruptions were permanent.

### Safety

A total of 74 patients experienced at least one toxicity event, for a total of 81 AEs. Of these, five were classified as haematological toxicities (3 anaemia of grade 2, 1 hypereosinophilia of grade 3, 1 neutropenia of grade 3), while the remaining were extra‐haematological toxicities. A total of 41 patients (42.7%) developed at least one irAE, for a total of 27 irAEs, leading to an incidence of 33.3%.

Four events were grade ≥3 (acute hepatitis, acute interstitial nephritis, elevation of amylase and lipase, neutropenia) and four classified as serious AEs (bronchiolitis, anaemia, acute hepatitis, elevation of amylase and lipase). No serious AE required hospitalization lasting more than 24–48 h, always as a precaution. Twenty‐four irAEs involved manifestations of extra‐haematological toxicity, while the remaining three events were haematological. Sixteen patients developed a single irAE, while the remaining six patients manifested two each. Twelve of the 27 irAEs (44.4%) involved the endocrine system. In particular, eight patients developed hypothyroidism; in two of these cases, an initial phase of thyrotoxicosis was observed, which later evolved into hypothyroidism. One patient developed isolated hyperthyroidism, that is, without subsequent evolution into hypothyroidism. All patients with thyroid hypofunction started indefinite replacement therapy with L‐thyroxine. Two cases of immune‐related skin toxicity (maculopapular skin reaction of grade 2 and erythematous‐oedematous lesions of the lower limbs of grade 2) have been recorded. Two cases of rheumatological irAEs have been documented: One patient developed rheumatoid arthritis (grade 2), while another patient presented with a scenario resembling polymyalgia rheumatica (grade 2). Regarding renal toxicities, two cases of acute interstitial nephritis have been recorded. Among the other irAEs reported, there is one case of lung toxicity, diagnosed as pneumonitis (grade 2); two cases of liver toxicity, characterized, respectively, by elevated transaminases (grade 1) and acute hepatitis (grade 3); one case of pancreatic toxicity, evidenced by increased pancreatic enzymes (grade 3). Additionally, two patients developed fever, while another experienced diarrhoea, both of grade 1. Finally, among the five haematological toxicities recorded, three were classified as irAEs: anaemia, hypereosinophilia and neutropenia (all grade 2). The first treatment approach was always with steroids, according to clinical practice. All irAEs resolved, except for the cases of hypothyroidism, managed and monitored through replacement therapy, one case of acute interstitial nephritis, characterized by a progressive deterioration of renal function despite treatment with corticosteroids, and one case of polymyalgia rheumatica, which persisted but with symptoms adequately controlled through steroid therapy. Early discontinuation of therapy compared to what was scheduled.

### Effectiveness and survival

The analysis of the best responses obtained from patients with irAEs showed that 30 patients achieved a CR or PR, while 11 patients showed SD or PD. In patients without irAEs, however, 42 obtained a CR or PR, and 13 had SD or PD. ORR was 73.2% in patients with irAEs and 76.4% in patients without irAEs. The comparison between the two groups did not reveal statistically significant differences (*p* = 0.157). At the end of the treatment period, the ORR was 48.8% in the group with irAEs and 41.8% in the group without irAEs. In particular, among patients with irAEs, 20 achieved a CR or PR and 21 achieved an SD or PD; while among patients without irAEs, 23 showed a CR or PR and 32 an SD or PD. No statistically significant differences emerged between the two groups (*p* = 0.5384, Table 2).

**TABLE 2:** Treatment response in the two study cohorts.

	irAE (*N* = 41)	No irAE (*N* = 55)	*p*
Best response, *n*			
CR/PR	30 (ORR 73.2%)	42 (ORR 76.4%)	0.157
SD/PD	11	13	
Final response			
CR/PR	20 (ORR 48.8%)	23 (41.8%)	0.5384
SD/PD	21	32	

Abbreviations: CR, complete response; irAE, immune‐related adverse event; ORR, overall response rate; PD, progression disease; PR, partial response; SD, stable disease.

**FIGURE 1:**
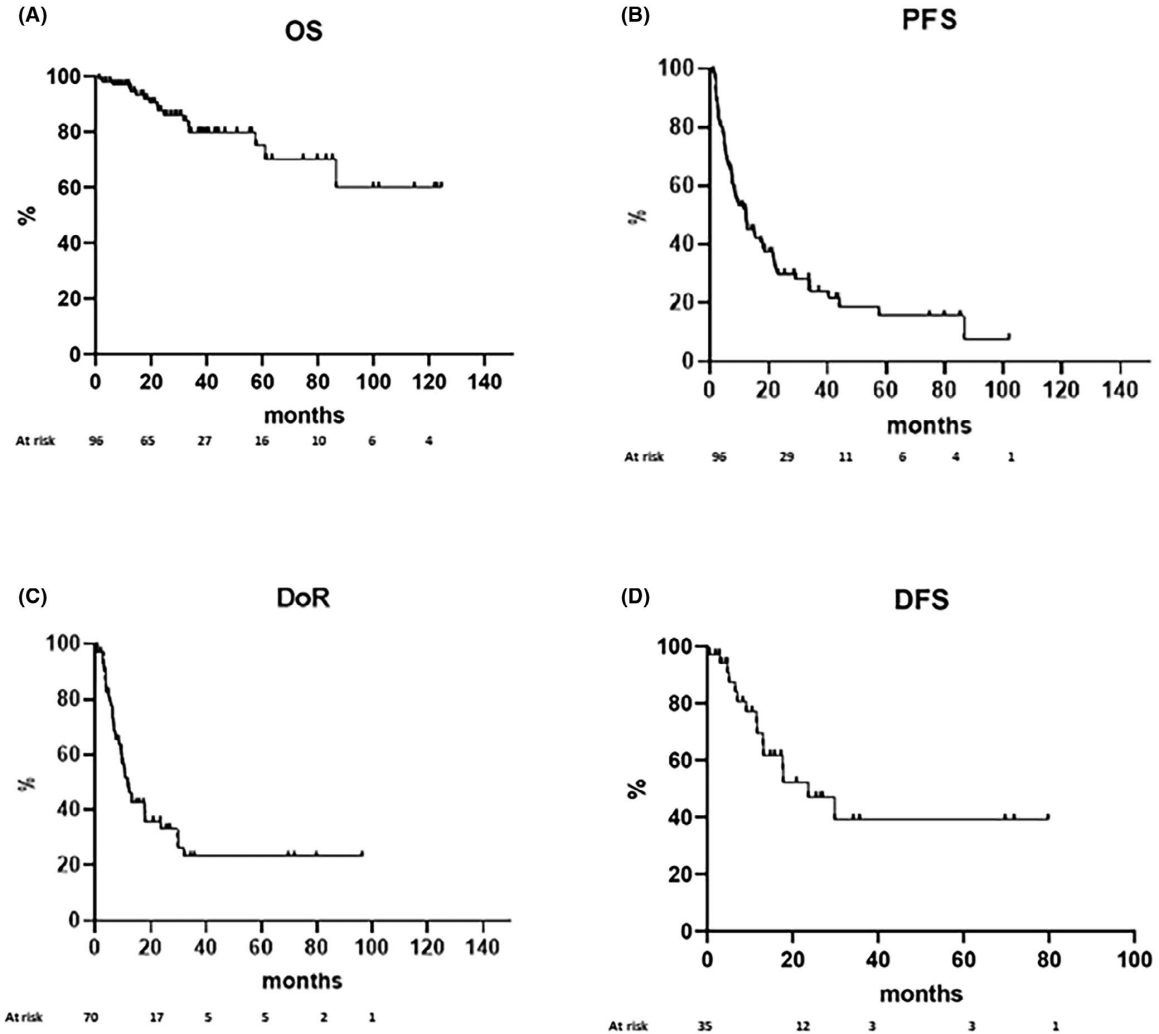
Survivals for the whole study sample. (A) Overall survival (OS). (B) progression‐free survival (PFS). (C) duration of response (DoR). (D) disease‐free survival (DFS).

Considering the whole study sample, with a median follow‐up of 33.6 months, the median OS has not been reached, indicating that a large portion of patients was still alive at the end of the observation period (Figure 1A). The median PFS was found to be 12.2 months (Figure 1B). Median DoR was 11.6 months (Figure 1C) and median DFS was found to be 23.7 months (Figure 1D).

**FIGURE 2:**
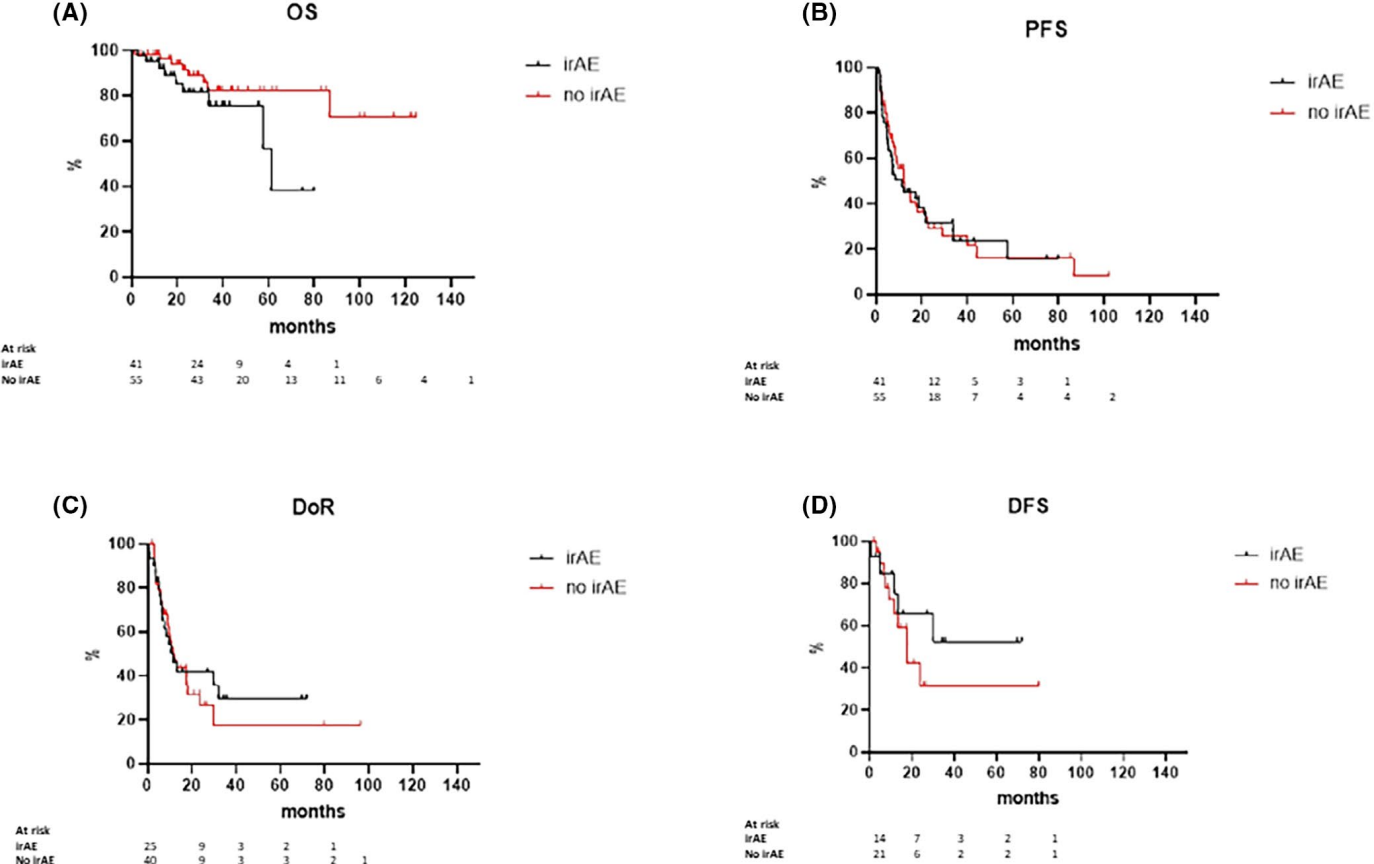
Survivals comparison for the two study cohorts: Patients who developed an immune‐related adverse event (irAE) and for who did not. (A) Overall survival (OS). (B) progression‐free survival (PFS). (C) duration of response (DoR). (D) disease‐free survival (DFS).

The survival analysis highlighted differences but not statistically significant (except for follow‐up) between patients who developed irAEs and those who did not. In fact, the median follow‐up was significantly shorter in patients without irAEs compared to those with irAEs (30.6 vs. 38.2 months, *p* = 0.0337). Patients who developed irAEs had a median OS of 61.1 months, while in patients without irAEs, the median OS has not yet been reached (Figure 2A). In patients with irAEs, the OS rates were 95.1% at both 6 and 12 months, 81.5% at 24 months and 75.7% at 36 months. In patients without irAEs, the OS rates were 98.2% at both 6 and 12 months; 88.9% at 24 months; and 82.4% at 36 months. The total number of recorded deaths/events was 17, especially nine in patients with irAEs and eight in patients who did not develop irAEs (*p* = 0.0923). No deaths were irAE‐related.

The median PFS in patients with irAEs was 11.37 months, while in patients without irAEs, it was 12.3 months (Figure 2B). In patients with irAEs, the PFS rates were 60.8% at 6 months, 44.9% at 12 months, 31.3% at 24 months and 23.5% at 36 months. In patients without irAEs, the PFS rates were 68.8% at 6 months, 49% at 12 months, 29% at 24 months and 21.5% at 36 months. Regarding PFS, the total number of events was 69: 29 in patients with irAEs and 40 in patients without irAEs (*p* = 0.8366). Regarding DoR, patients who developed irAEs showed a median of 11.7 months, while in patients without irAEs, the median DoR was 11.6 months (Figure 2C, *p* = 0.9238). Finally, DFS did not show any statistically significant difference between the two groups. Specifically, in patients who developed irAEs, the median DFS was not reached, while in patients without irAEs, it was 17.8 months (Figure 2D, *p* = 0.2815).

## DISCUSSION

The introduction of drugs belonging to the class of ICIs has represented a significant therapeutic advance in the treatment of R/R HL. However, their use is associated with the occurrence of irAEs, the prognostic relevance of which has not yet been definitively clarified in patients with HL.^23^, ^25^, ^26^, ^27^ In various oncological contexts, irAEs occurrence has been correlated with improved outcomes, but this association has not been confirmed in patients with HL as literature data are very poor.^25^, ^26^, ^27^ Our observational retrospective/prospective study aimed to fill this literature gap. The results obtained contributed to broaden the role of irAEs in this patient population and suggested insights for future studies aimed at identifying potential predictive biomarkers of response and clarifying the immunological mechanisms involved in the efficacy of ICIs in HL. In particular, results showed that the presence of irAEs does not seem to impact on treatment outcomes or survivals significantly. First, the analysis of treatment responses revealed a similar ORR in patients with and without irAEs (73.2% vs. 76.4%, respectively), with a *p*‐value greater than 0.9999, suggesting that the presence of irAEs does not influence the likelihood of achieving an initial response to treatment with ICI. However, despite the non‐statistically significant test results, the percentage of patients who maintained a CR or a PR was substantially higher in patients with irAEs (48.8%) than in patients without irAEs (41.8%).

Present data suggested that, although irAEs may be indicators of immune system activation, they do not seem to be correlated with a greater or lesser likelihood of obtaining a lasting response to treatment. However, some insights coming from our analyses lay the basis to further explore the relationship between irAEs and response duration, considering possible subgroups of patients with specific clinical or biological characteristics.

Regarding survival indicators, patients with irAEs showed a median OS of 61.1 months, while that of the group without irAEs was not reached (*p* = 0.2048). These results suggest that the development of irAEs is not an independent prognostic factor for the OS of patients, contrary to what has been observed in other contexts, where the occurrence of irAEs has sometimes been associated with better treatment response and increased survival.^30^ A meta‐analysis evaluated whether irAEs could serve as surrogate end‐points for OS in patients treated with ICI for several advanced tumours.^31^ The results of this meta‐analysis showed weak correlations between the rate of irAEs and OS, suggesting that the variability in survival is only partially explained by irAEs.^31^ Factors, such as the expression of PD‐L1 and the mutational burden, may be more relevant. Additionally, severe irAEs can reduce the efficacy of therapy due to treatment interruptions but for the results of the meta‐analysis, the rate of irAEs cannot be considered a reliable surrogate for OS.^31^ Further studies on individual data are needed to explore this association and the prognostic role of irAEs in patients treated with ICIs.^31^

The lack of association between the development of irAEs and the treatment response observed in our study may reflect the specificity of HL as a disease, whose biological and clinical peculiarities could influence the efficacy of ICIs. The analysis of PFS showed a median of 11.37 months in patients with irAEs, compared to 12.3 months in patients without irAEs, with similar PFS rates at 6, 12, 24 and 36 months in both groups. Again, the comparison between the survival curves revealed no statistically significant differences (*p* = 0.8366), suggesting that the presence of irAEs does not significantly affect the duration of disease control. However, median DFS was not reached in patients who developed irAEs.

Nevertheless, it is likely that the association between irAEs and the duration of disease control may vary based on the type of cancer and the therapy used, as demonstrated by other studies.^32^

Regarding safety, only one irAE (acute interstitial nephritis) caused permanent treatment discontinuation, supporting the manageability and tolerability of this particular type of AEs. Overall, the results of our study suggest that the presence of irAEs does not have a particular significant impact on treatment response outcomes and survival indices in patients with HL treated with ICIs. The results of our study are in agreement with those of the study by which evaluated the incidence and outcomes of irAEs after the use of ICIs in patients with HL.^33^ According to the latter study, after matching for propensity score of known prognostic factors for HL, no significant difference in OS emerged between patients with or without irAEs (hazard ratio 0.84; 95% CI 0.57–1.23; *p* = 0.36). Furthermore, no significant differences in OS were found among the various subtypes of irAEs. The authors conclude that irAEs are common in patients with HL treated with ICIs, but most of these patients were able to continue treatment with ICIs without the development of irAEs influencing the outcome.

The present report is the second part of a wider study: Results focused on the impact of irAEs in patients with R/R HL treated with ICI can be compared to what was reported in the NHL study.^24^ These results suggest that the incidence of irAEs might be associated with a trend towards better long‐term survival in patients with NHL treated with ICIs, although the observed differences were not statistically significant. Regarding the frequency of the various types of irAEs, the study results on NHL show that the most common irAEs were those involving the endocrine glands, particularly the thyroid.

The limitations of our study must be considered for a correct interpretation of the results obtained. Since this is an observational study, it is not possible to establish a causal relationship between irAEs and clinical outcomes; indeed, the observed associations could be influenced by unconsidered confounding factors. Moreover, although the median follow‐up is 33.6 months, a longer observation period may be necessary to fully observe long‐term effects, such as OS and PFS, which may further evolve over time. Additionally, the diagnosis and classification of irAEs are complex and subject to variability among physicians, which may have affected the accurate assessment of these events.^34^ These limitations indicate the need for further studies with larger samples and longer follow‐ups to confirm the results obtained and deepen the dynamics between irAEs and the efficacy of treatment with ICI in HL as for haematological diseases literature is still poor.^34^

The complexity of irAEs and the variability in response to immunotherapeutic treatments require further studies, possibly on large cohorts of patients, to better explore the relationship between irAEs and response duration, as well as to identify any subgroups of patients who might benefit more from immuno‐oncological treatment. In the future, it may be useful to explore the biological and molecular bases that could explain the variability of the response to ICIs in patients with and without irAEs, with a particular focus on predictive response biomarkers and the underlying immunological mechanisms.

## AUTHOR CONTRIBUTIONS

LA, VS and PLZ designed the study, performed data curation, writing—original draft, writing—review and editing and supervised the project. LA performed statistical analyses. PLZ was responsible for funding acquisition. BC, VLP, AB, UP, CP and AB contributed to collect the data, contributed to putting together and interpreting the figures and tables and gave feedback on the manuscript writing—review and editing.

## FUNDING INFORMATION

The work reported in this publication was funded by the Italian Ministry of Health, RC‐2025‐2797392 project.

## CONFLICT OF INTEREST STATEMENT

None of the authors declare any potential conflict of interest.

## ETHICS STATEMENT

The study was approved by our local Ethical Committee (Comitato Etico Area Vasta Emilia Centrale di Bologna, IRCCS Azienda Ospedaliero‐Universitaria di Bologna, approval id code 730/2019/Oss/AOUBo) and registered in the Italian Registry of Observational Studies.

## CONSENT

Patients were included after written informed consent. Patients no longer alive at the time of study were included in the study, according to the study permit from the Ethical Review Authority.

## Data Availability

The data of this study are publicly available at the Zenodo platform (https://doi.org/10.5281/zenodo.17278831).
